# Modeling of RAS complexes supports roles in cancer for less studied partners

**DOI:** 10.1186/s13628-017-0037-6

**Published:** 2017-08-11

**Authors:** H. Billur Engin, Daniel Carlin, Dexter Pratt, Hannah Carter

**Affiliations:** Division of Medical Genetics, Department of Medicine, Universsity of California, San Diego, 9500 Gilman Dr., La Jolla, CA 92093 USA

**Keywords:** RAS, Hypervariable region, Allosteric region, Effector lobe, Protein-protein interaction, Protein interaction interface, Protein structure, Cancer, Tumor suppressor interaction

## Abstract

**Background:**

RAS protein interactions have predominantly been studied in the context of the RAF and PI3kinase oncogenic pathways. Structural modeling and X-ray crystallography have demonstrated that RAS isoforms bind to canonical downstream effector proteins in these pathways using the highly conserved switch I and II regions. Other non-canonical RAS protein interactions have been experimentally identified, however it is not clear whether these proteins also interact with RAS via the switch regions.

**Results:**

To address this question we constructed a RAS isoform-specific protein-protein interaction network and predicted 3D complexes involving RAS isoforms and interaction partners to identify the most probable interaction interfaces. The resulting models correctly captured the binding interfaces for well-studied effectors, and additionally implicated residues in the allosteric and hyper-variable regions of RAS proteins as the predominant binding site for non-canonical effectors. Several partners binding to this new interface (SRC, LGALS1, RABGEF1, CALM and RARRES3) have been implicated as important regulators of oncogenic RAS signaling. We further used these models to investigate competitive binding and multi-protein complexes compatible with RAS surface occupancy and the putative effects of somatic mutations on RAS protein interactions.

**Conclusions:**

We discuss our findings in the context of RAS localization to the plasma membrane versus within the cytoplasm and provide a list of RAS protein interactions with possible cancer-related consequences, which could help guide future therapeutic strategies to target RAS proteins.

**Electronic supplementary material:**

The online version of this article (doi:10.1186/s13628-017-0037-6) contains supplementary material, which is available to authorized users.

## Background

Tumor exome sequencing studies have uncovered RAS mutations in over 30% of solid tumors, yet to date no therapy has been discovered to effectively treat RAS driven cancers. RAS proteins themselves provide an attractive therapeutic target but efforts aimed at drugging RAS directly thus far have failed [1]. More complete characterization of RAS signaling may provide badly needed insights to support renewed efforts aimed at developing RAS-targeted therapies [1, 2]. In particular, little is known about how RAS proteins interact with less-studied interaction partners, and how these proteins contribute to tumorigenesis.

**Fig. 1:**
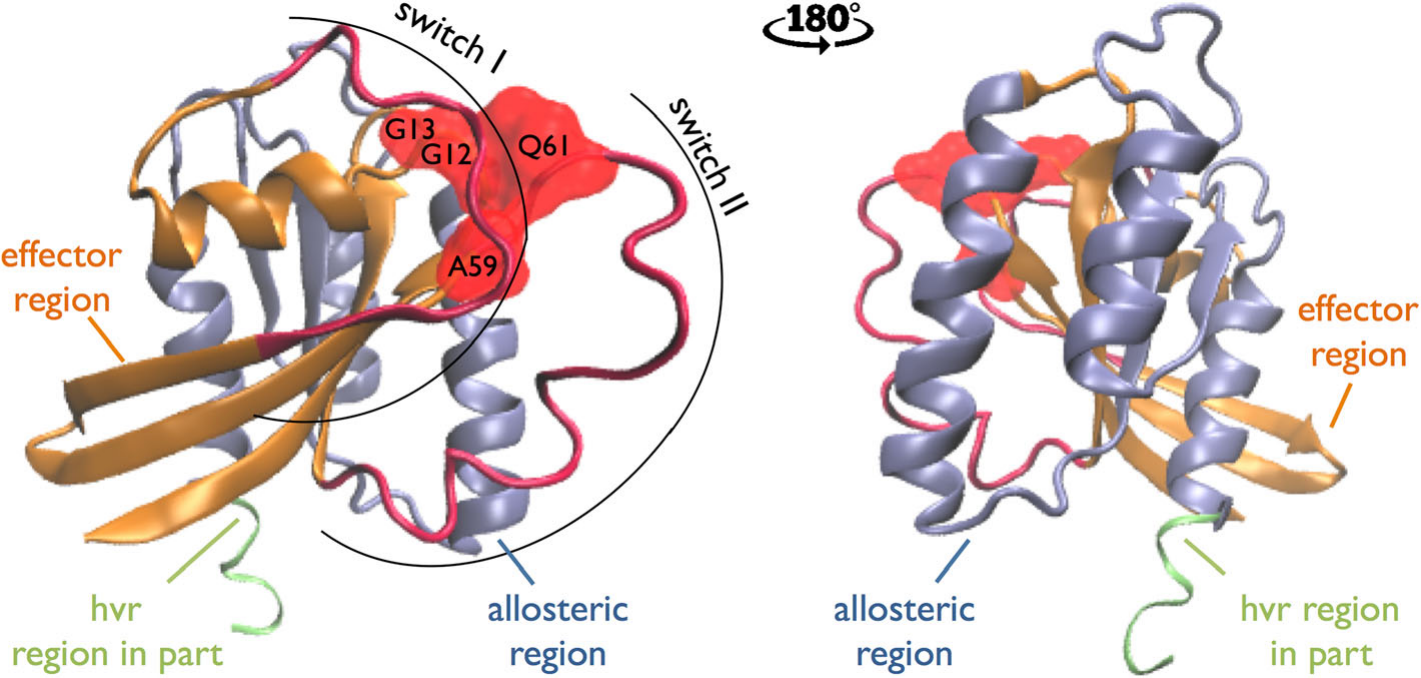
3D structure of HRAS (PDB ID: 6Q21:A). Effector region is in *orange*, allosteric region is in *blue* and the switch regions are depicted in *red*. The G12, G13, A59 and Q61 hotspot mutations are illustrated in *red*. The HVR region is partially (residues 166 to 171) present in the PDB and the figure

RAS is a small GTPase that localizes to the plasma membrane (PM) and upon receiving extracellular stimuli, initiates multiple signaling cascades inside the cell [3]. There are 4 major isoforms of RAS, including KRAS4A, KRAS4B, HRAS and NRAS. All RAS isoforms transition between active and inactive states, a process that is regulated by guanine nucleotide exchange factors (GEFs) and GTPase activating proteins (GAPs). The isoforms are highly similar in amino acid sequence; all isoforms are 188–189 amino acids in length, with nearly 85% sequence identity [4]. The first 87 residues are 100% homologous and most of the sequence differences occur in the short C-terminal hypervariable region (HVR) (residues 166 to 189), which is only 10% conserved among RAS proteins (Fig. 1).

RAS signaling is deceptively complex. In contrast to their sequence similarity, different RAS isoforms have been associated with distinct functionality and show distinct patterns of expression in various cell lines and tissues [5, 6]. Recent work has shown that RAS isoforms also form distinct homo- [7] and hetero-dimers [8]. In cancer, the clinical phenotype of patients with RAS mutations is highly context dependent. The prevalence of specific mutant RAS alleles differs among tumor types, as does the predominantly mutated RAS isoform. For example, head and neck [9] tumors carry mutant HRAS, melanomas [10] carry mutant NRAS and pancreatic tumors [11] carry mutant KRAS. For KRAS mutant driven tumors, non-small cell lung cancers tend to carry G12C mutations [12], while the G12D [13] substitution is predominantly found in pancreatic cancer. This complexity suggests that no single therapy will be able to universally target mutant RAS.

Recently, Chavan et al. [14] described a mechanism by which G12 mutations render RAS isoforms more active. According to this model, the G12 residue binds to the HVR, keeping RAS in a less active state by blocking interaction of RAS with effectors. Mutations at this site may impair HVR binding, increasing RAS interaction with downstream effectors. Nonetheless, differences in the prevalence of G12 amino acid mutants across tumor types cannot be explained by the weakening of the HVR binding by G12 mutations alone. If that were the case, the mutant amino acids should occur with the same frequency across tumors. One possible explanation for the difference in mutant prevalence is that G12 mutations also affect RAS binding with protein interaction partners, the expression of which may differ by tumor type [15]. Thus better understanding of RAS protein interactions will be essential for understanding its role in tumorigenesis.

From a therapeutic perspective, RAS proteins have been perceived as “undruggable” due to a lack of effective inhibitors, even after more than three decades of research [16]. However, very recently four groups have reported novel allosteric and orthosteric inhibitors targeting RAS protein protein interactions (PPI) by taking advantage of a pocket that becomes accessible upon compound binding [17–20]. In 2016, Athuluri-Divakar et al. [21] identified a high affinity small molecule inhibitor that binds to RAS effectors such as RAF, Ral-GDS and PI3Ks and disrupts their interactions with RAS. Subsequently, RAS inactivation through PPI disruption has become the leading approach for targeting RAS [22], further motivating studies of the RAS protein interaction network.

RAS PPIs have predominantly been studied in the context of the RAF and PI3kinase oncogenic pathways [23] and structural analyses have concluded that RAS isoforms bind to canonical downstream effectors and upstream regulators in these pathways using the highly conserved switch I (residues between 30 and 40) and II regions (residues between 60 and 76, [24]). Understanding of RAS interactions is largely limited to the interactors that have RAS binding related domains (RBrDs) (see Methods), here referred to as “canonical interactors”. Other, non-canonical RAS interactors have been experimentally identified, including FNTA, FNTB and chaperone proteins PDE6D and LGALS1, but it remains poorly understood how these proteins interact with RAS.

To study RAS isoform specific PPIs, including partners with no well-defined RBrD, we predicted which residues on the surface of RAS interact with each binding partner. The resulting map of binding to RAS was used to model possible RAS complexes and identify possible competitive binding relationships. We then analyzed the putative effects of mutant alleles on RAS interactions with binding partners. Our analysis suggests a novel binding interface on the allosteric region/HVR of RAS proteins that is accessible when RAS is not localized to the plasma membrane. Interestingly, multiple interaction partners that bind via this new interface are putative cancer genes.

## Results

**Fig. 2:**
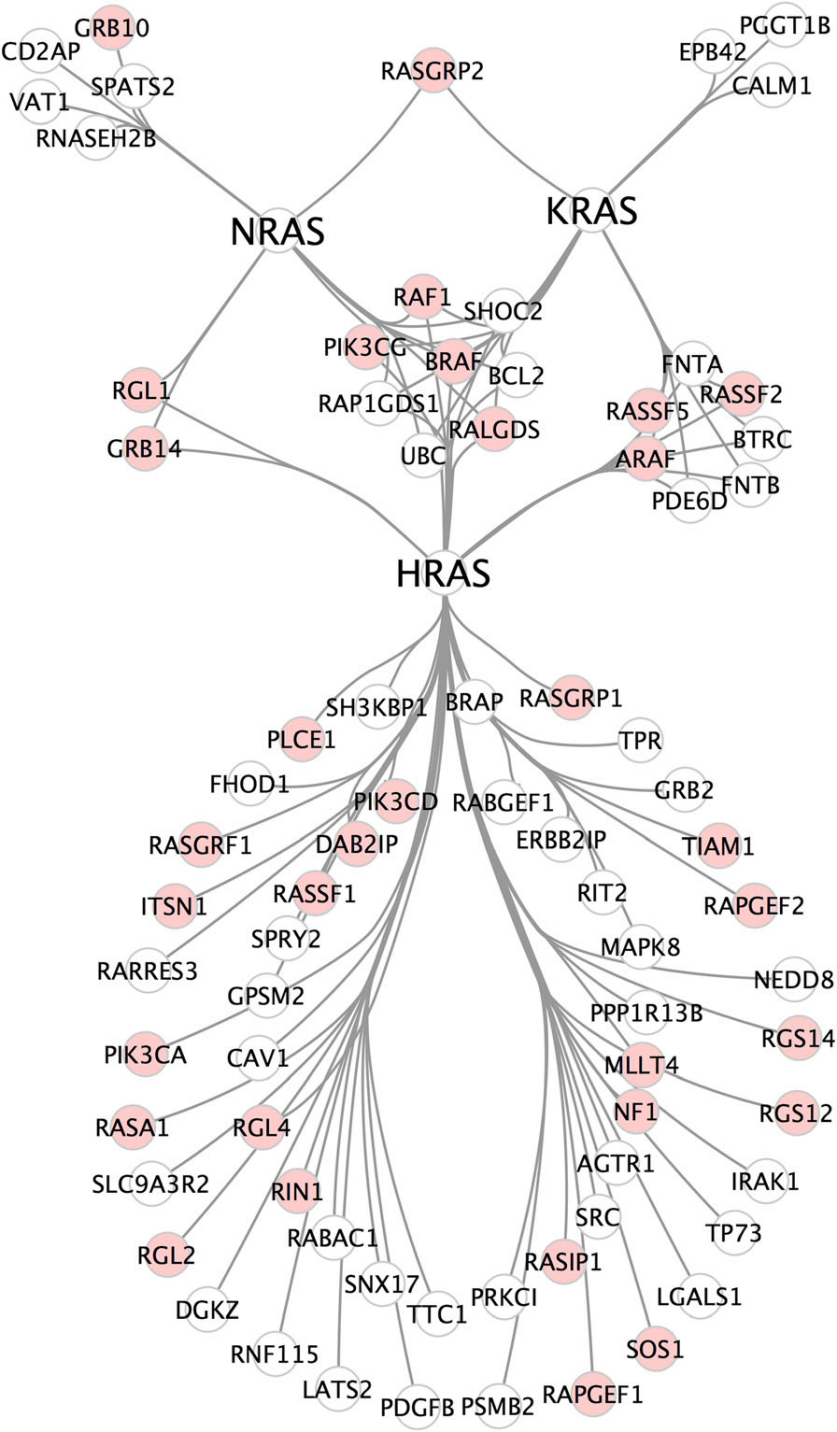
Literature curated RAS isoform specific PPI network. Nodes represent proteins and edges represent physical interactions between RAS isoforms and binding partners that have direct experimental evidence in the literature. The proteins with an RBrD are shown in *pink*

In order to identify interaction interfaces on RAS proteins, we first curated an experimentally verified RAS isoform specific PPI network that consists of as many as 103 RAS protein interactions (see Methods). Of 77 RAS interacting proteins in our network, 45 proteins do not have an RBrD (Fig. 2). We first curated interface residues from seven RAS co-complex structures available in the Protein Data Bank [25] (PDB; 6 HRAS, 1 KRASs and no NRAS complexes), all of which capture canonical RAS interactors.

**Fig. 3:**
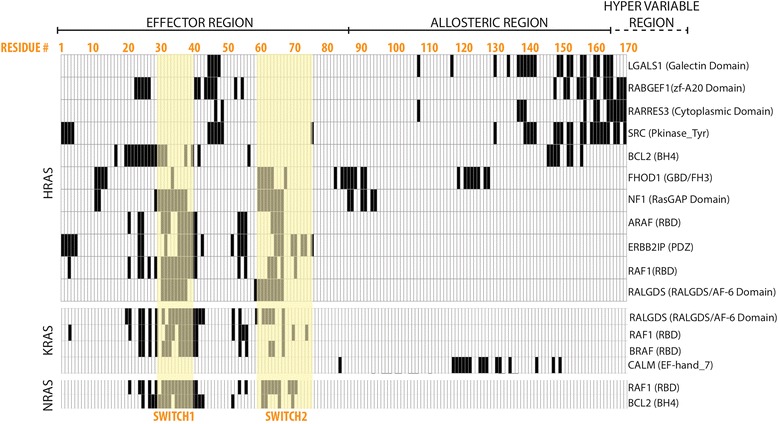
A map of the RAS amino acid residues predicted to mediate physical interactions with RAS binding partners. HRAS, KRAS and NRAS are each depicted by separate matrices. Each row corresponds to a binding partner, and each column corresponds to an amino acid position on a RAS protein. If a residue is involved in a specific PPI, the corresponding cell is painted in *black*. Amino acid residue position numbers are displayed in *orange* at the top of the figure. The residues comprising the effector, allosteric and hypervariable regions are labeled. The residues in the switch regions are highlighted in *yellow*. The domain on the interaction partner that is predicted to interact with RAS is listed in parentheses next to the binding partner’s name

**Fig. 4:**
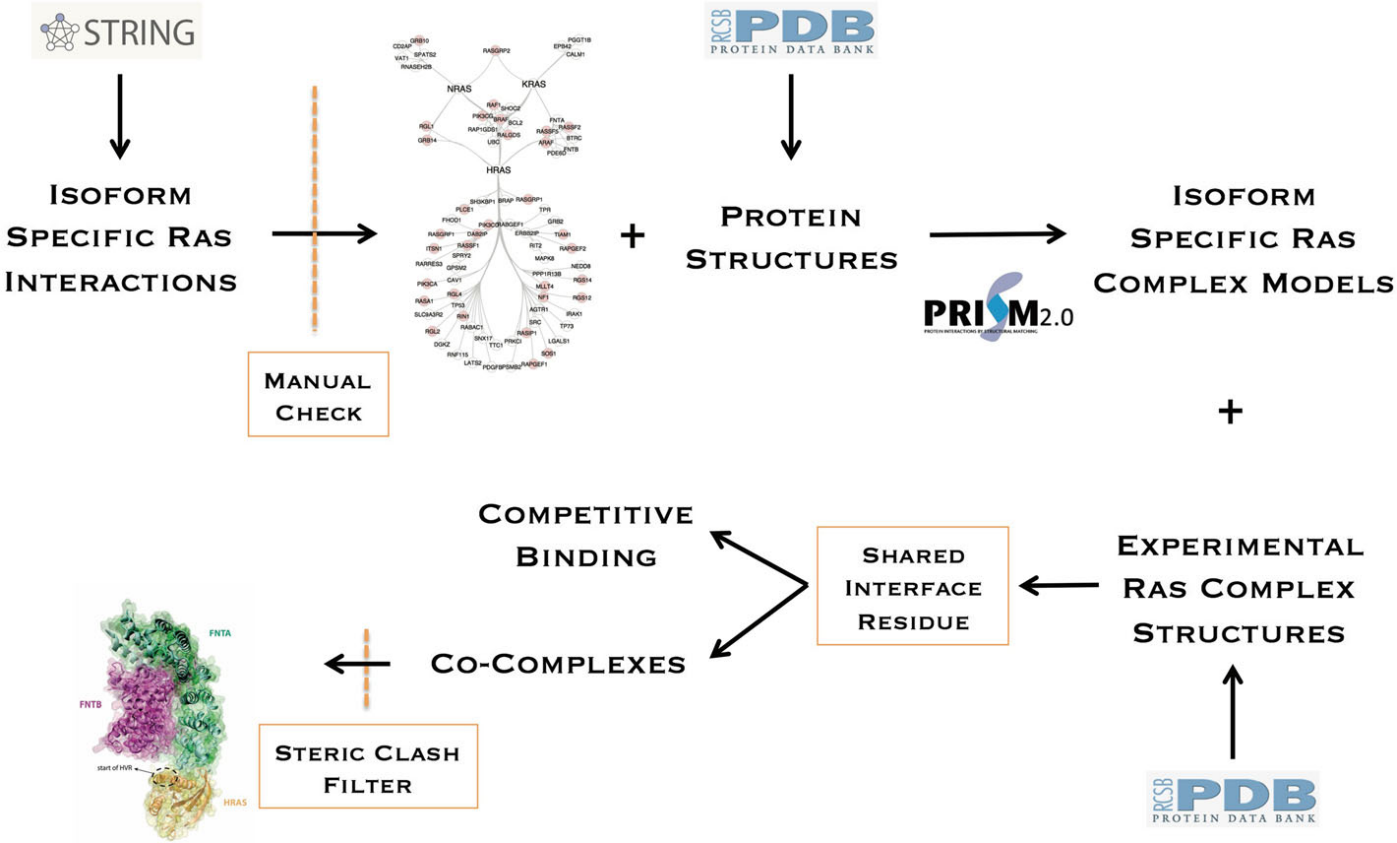
Description of the pipeline for determining multi-protein RAS complexes. Protein interactions in STRING were manually evaluated and PDB structures were acquired. PRISM was used to predict complex formation. These complexes were combined with available RAS complexes in the PDB to gain a complete view of RAS binding surfaces. This was used to determine which binding partners could bind simultaneously. Simultaneous binding was then assessed in a 3D model to ensure that no steric clashes resulted

We next built RAS 3D complex models using PRISM [26], a template-based protein complex prediction server that has been extensively benchmarked and reports high accuracy for identifying interfaces [27]. PRISM predicted binding interfaces for 20 complexes involving the RAS proteins and 17 binding partners. PRISM was not able to identify binding interfaces for the other 37 partners with available structural data including ITSN1, RGS12, GRB1 and UBC. We used the 20 models of RAS interactions to determine which amino acids on the surface of RAS participate in each interaction (see Methods). This resulted in a map of interface residues on RAS proteins (Fig. 3) enabling us to analyze patterns of interface residue usage across interaction partners (Fig. 4). This map revealed two categories of RAS interaction partners: proteins that bind via the switch regions, and proteins that bind via the allosteric region and/or the HVR. The HVR does not adopt a stable conformation and therefore is challenging to capture via X-ray crystallography. Given that the available PDB structures in general do not include the full HVR, PRISM predictions for binding in the allosteric/HVR region are best interpreted as “interactors that do not bind to the switch region”.

### RAS binding partners that do not bind to the switch region

Most studies of RAS signaling in cancer focus on effectors that bind the switch regions, with few studies addressing binding partners without RBrDs. Among these, one of the earlier studies addresses the involvement of the HVR in the KRAS – CALM interaction [6]. This result has only recently been revisited [2, 14] after the discovery of RAS homodimerization [28]. It has previously been demonstrated that residues mediating RAS dimerization, R135, R161 and R164 located in the 4th and 5th helices, are not in the effector region. These helices are conserved across all three of the RAS proteins [29]. A different line of evidence for the non-switch regions’ involvement in RAS interactions is present in the PDB complex between PDE6D and RHEB (PDB ID: 3T5G). RHEB is a GTPase with 80% sequence similarity to RAS proteins. RHEB primarily localizes to the ER and Golgi Apparatus [30] and according to a co-crystal structure (PDB ID: 3T5G) it interacts with chaperone protein PDE6D via its HVR. PDE6D is also reported to act as a chaperone for RAS isoforms, and most likely interacts with them via their HVR region as well.

Interaction partners that do not contain an RBrD [31] and are predicted to bind RAS proteins outside of the switch region include RABGEF1, LGALS1, RARRES3, SRC (HRAS) and CALM (KRAS). We further investigated published data on these RAS interactions to determine their effects on RAS signaling. Interestingly, many of these genes are determinants of RAS protein localization, thereby directing signaling toward or away from canonical RAS effector pathways.

Rab5 GDP/GTP exchange factor (RABGEF1) acts as an E3 ligase for HRAS, and HRAS monoubiquitination promotes its localization to the endosome, thereby decreasing ERK signaling [32]. RABGEF1 possesses a zinc finger (ZnF) domain similar to that of A20 with E3 ligase activity [33–35], and Xu et al. [32] suggested that the interaction between RABGEF1 and HRAS occurs via this domain. A mutation in the ZnF domain was reported to obstruct RABGEF1’s ability to ubiquitinate RAS, providing support for ZnF-mediated RAS binding.

LGALS1 is a cytosolic protein that has a dual role in RAS biology: it acts both as a chaperone that transports depalmitoylated HRAS to the Golgi and a critical scaffolding protein [36] that is essential for RAS PM association [37]. It has been shown that the RAS inhibitor Farnesylthiosalicylic acid (FTS) simultaneously disrupts RAS PM localization [38] and HRAS interaction with LGALS1 [37]. FTS associates with RAS via the HVR region, and its perturbation of the HRAS-LGALS1 interaction is consistent with our prediction that LGALS1 interacts with the HVR.

The inhibitory interaction between retinoic acid receptor responder protein 3 (RARRES3) and HRAS occurs in the Golgi [39, 40]. RARRES3 interaction is reported to suppress EGF-induced RAS activation by decreasing the plasma membrane localization of RAS [40]. The RARRES3 protein lacks well-defined domains, however there are three partitions of its sequence: cytoplasmic, trans-membrane and lumenal. Our interface region predictions implicate the cytoplasmic region of RARRES3 as binding to RAS.

SRC is a proto-oncogene that acts upstream of RAS [41], and activates the oncogenic RAS pathway by phosphorylating SHC [42] in tumor cells. HRAS is also reported to directly interact with SRC and negatively regulate its kinase activity; when bound to HRAS, SRC auto-phosphorylation and phosphorylation of its target NR2A are inhibited in the brain [43]. Interestingly, in contrast to other kinases like RAFs, which have RBrDs, SRC binds to HRAS via its kinase domain. Both HRAS and SRC are known for their oncogenic activity, thus the inhibitory activity of HRAS toward SRC suggests tumor suppressive activity. Impaired HRAS signaling, whether due to mutation or therapeutic intervention, could reduce its inhibition of SRC, thereby promoting SRC’s oncogenic activity.

CALM is thought to specifically bind KRAS-4B among RAS family proteins [6, 8, 44, 45]. This interaction regulates KRAS membrane association [46], which is crucial for RAS activity in cancer. CALM translocates KRAS from the PM to the cytosol by sequestering its farnesyl group, thereby regulating the concentration of KRAS at the PM [47]. In a range of cell lines, inhibition of CALM induced KRAS and RAF-1 activity [48]. CALM is known to bind KRAS in a RAF/switch region independent manner [8] and the HVR is involved in this interaction [2, 6]. Our model suggested that CALM binds HRAS in the allosteric region. Given the absence of the HVR from existing HRAS crystal structures, it is unclear whether CALM actually contacts the allosteric region as well as HVR, or whether building is mediated exclusively by the HVR. Nonetheless, our predictions are consistent with reports that CALM does not bind the switch region.

**Fig. 5:**
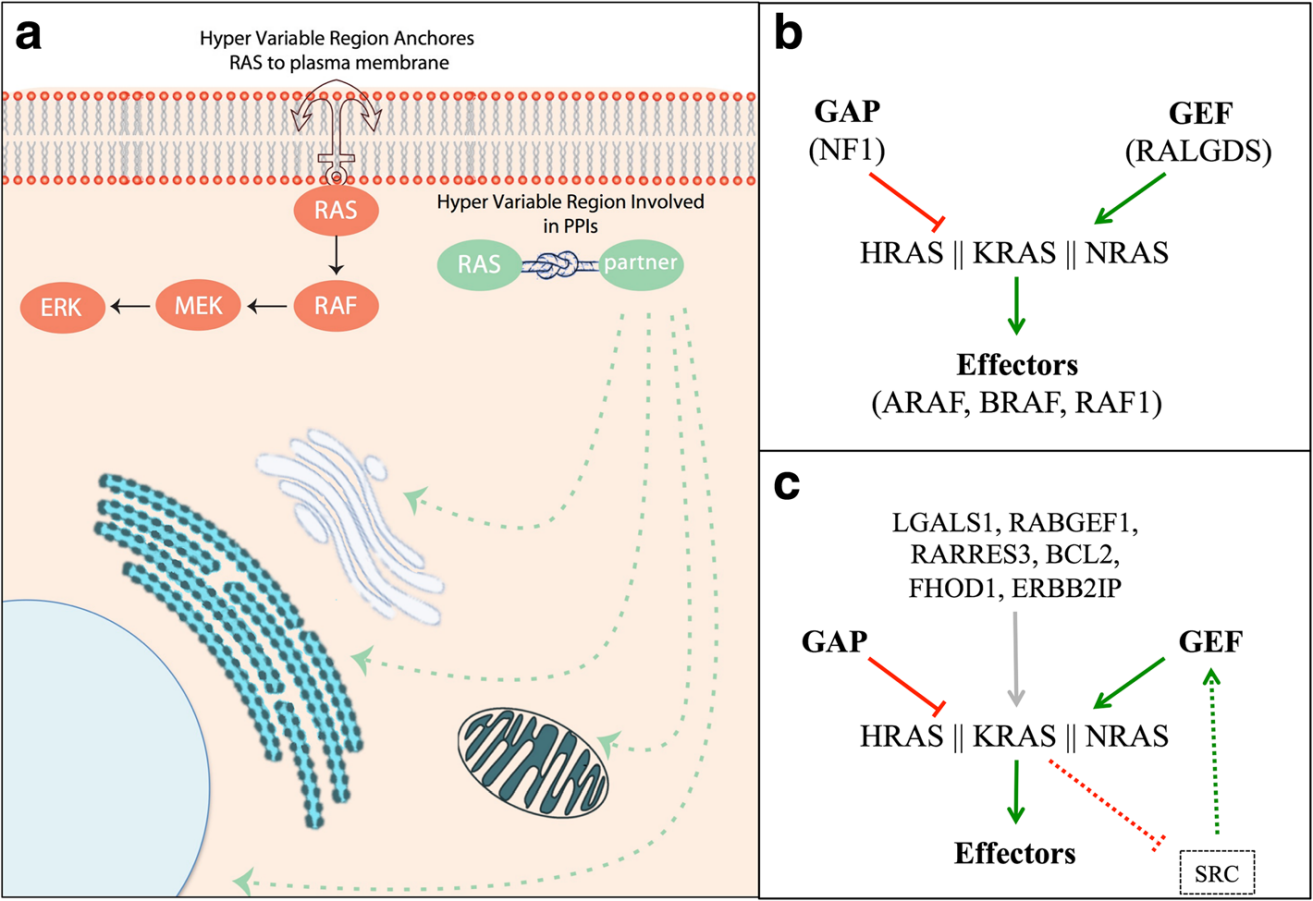
HVR and sub-cellular localization of RAS. **a** Our structural models suggested the involvement of the RAS allosteric region and HVR in protein complex formation. According to the evidence in the literature we hypothesize that such interactions most likely occur when RAS is not anchored to the PM. Our structural models (**b**) recapitulate the well-established RAS interactors that host an RBrD, (**c**) and suggest additional interactions that regulate oncogenic RAS signaling

Based on this evidence and our structural predictions, we propose that when RAS is not localized to the PM, the allosteric and the HVR regions of RAS proteins are involved in interactions with proteins that regulate RAS localization and PTMs. Interestingly, some of these partners are reported to suppress tumorigenic RAS signaling, including RARRES3 [49] and RABGEF1 [50], CALM [51], while others such as LGALS1 [52] and SRC [53] are reported to promote it (Fig. 5). Thus non-switch mediated interactions of RAS may play a significant role in RAS’s oncogenic potential.

We next compared PRISM’s interface to interface predictions made by similar algorithms including pyDock [54], ZDOCK [55] and COTH [56]. For this comparison, we selected two predictions outside of the Switch regions (HRAS – RARRES3 and KRAS - CALM), and one interaction known to use the Switch region (HRAS – RAF1). While there was some disagreement on the specific residues involved, all methods agreed on whether or not Switch region residues were involved in the interaction (Additional files 1, 2 and 3: Figures S1, S2 and S3). For example, PRISM predicted that RARRES3 does not bind the HRAS switch regions, but instead binds predominantly to residues in the allosteric and hyper-variable regions. The other three methods also predicted complexes that did not involve residues in the switch region (with the exception of pyDock which included 3 residues from one Switch motif out of 16 total residues at the predicted binding interface), and implicated multiple residues in the allosteric region (Additional file 1: Figure S1). All methods are capable of returning no result if there is no favorable binding interface. Altogether, this provides strong evidence that RARRES3 interacts with HRAS through an interface that does not involve the canonical switch region binding site.

Finally, we investigated the possibility that proteins binding to RAS isoforms outside of the switch region use a common structural motif. None of the proteins interacted with RAS using the same domain. We next used VAST to assess pairwise structural similarity of these interfaces using the PDB IDs listed in Table 1. This analysis did not identify common structure among interfaces binding outside of the switch region. This may suggest that the disordered HVR alone, or in combination with the allosteric region, allows interaction with multiple distinct structures. VAST did report similarity of binding interfaces on BLC2, NF1 and RALGDS, all of which bind at least partially to the switch region but none of which have an RBrD*.*

**Table 1:** List of RAS isoform specific complex models generated via PRISM

PDB Chain for the RAS Protein	Gene Symbol of the RAS Protein	Interaction Partner	Gene Symbol of the Interactor Protein	Interface	Energy Score	Pfam Domain/Family of the Interactor
NRAS_model	NRAS	4g0nB	RAF1	1c1yAB	−52.35	RBD
NRAS_model	NRAS	4g0nB	RAF1	1c1yAB	−34.05	RBD
NRAS_model	NRAS	1g5mA	BCL2	1u8yAB	−23.32	BH4
4dsoA	KRAS	4g0nB	RAF1	1c1yAB	−46.71	RBD
4dsoA	KRAS	1c1yB	RAF1	1c1yAB	−45.94	RBD
4dsoA	KRAS	3kh0B	RALGDS	1lfdCD	−28.69	RALGDS/AF-6
4dsoA	KRAS	3ny5A	BRAF	1c1yAB	−26.42	RBD
4dsoA	KRAS	1zuzA	CALM	1qs7AC	−24.38	EF-hand-7
6q21A	HRAS	2lktA	RARRES3	3hbrAB	−38.81	Cytoplasmic Domain
6q21A	HRAS	4g0nB	RAF1	1c1yAB	−36.18	RBD
6q21A	HRAS	3kh0B	RALGDS	1lfdCD	−35.49	RALGDS/AF-6
6q21A	HRAS	1nf1A	NF1	1tx4AB	−28.38	RasGAP
6q21A	HRAS	3dadA	FHOD1	2ghpBD	−27.8	GBD/FH3
6q21A	HRAS	3w58C	LGALS1	2wfhAB	−26.46	Galectin
6q21A	HRAS	2h3lA	ERBB2IP	1w9aAB	−25.15	PDZ
6q21A	HRAS	2qbwA	ERBB2IP	3e9mAD	−23.61	PDZ
6q21A	HRAS	2c7nI	RABGEF1	2y3gAD	−23.53	zf-A20
6q21A	HRAS	1wxmA	ARAF	1c1yAB	−23.5	RBD
6q21A	HRAS	1g5mA	BCL2	1t35EF	−22.79	BH4
6q21A	HRAS	1yojA	SRC	1xdkEF	−21.72	Pkinase_Tyr

### Implications of somatic hotspot mutations for RAS interactions

RAS mutations are most commonly observed at residues 12, 13, 59 and 61 in tumors and these mutants have all been reported to favor RAS’s GTP-bound state. For KRAS, 80% of mutations occur at the 12th amino acid, while for NRAS, 60% of mutations affect residue Q61. HRAS, on the other hand, displays a less biased distribution with 50% and 40% of mutations occurring at codons 12 and 61 respectively [57]. In vitro studies have associated G12C mutations with the formation of bulky DNA adducts caused by carcinogens in tobacco smoke [58] and lung cancers are enriched for this specific mutation [57]. Studies of carcinogenesis have also revealed that UV-radiation causes pyrimidine dimers and this process can generate RAS Q61 mutations, which are prevalent in melanoma [59]. Colorectal cancers and haematopoietic/lymphoid cancers harbor codon 13 mutations at high frequency. Thus mutational signatures associated with different exposures and etiologies contribute to tumor-type specific differences in RAS codon mutation prevalence. Different codons are also associated with clinical characteristics of patient tumors. For example, colorectal cancer patients with codon 13 mutations respond to treatment with anti-EGF receptor cetuximab-based therapy, however, patients with mutations at G12 do not [57]. We therefore sought to determine whether specific PPIs could be affected differently by different codon mutations.

We used FoldX [60] to determine the effects of RAS mutations on binding to interaction partners using the new protein interaction models. FoldX has previously been applied to analyze the consequences of cancer mutations affecting the RAS/MAPK pathway [61]. Cancer mutations that were assigned destabilizing energies by FoldX were found to occur in regions of RAS proteins associated with RAS activation, with few predicted to impair protein folding. We adopted a similar approach to examine common effector region mutations (G12, G13, A59 and Q61, 57] and two recently reported mutation hotspots (K117N and A146T) in the allosteric region [62] (Table 2). Our RAS complex models did not predict direct binding to RAS K117 or A146 residues, however BCL2 was predicted to bind a neighboring surface region on HRAS that spans the residues 147–150.

**Table 2:** FoldX predictions of RAS mutation effects for RAS complexes

RAS Isoform	Interaction Partner	Mutation	Effect
HRAS	BCL2	A146T	stabilizing
HRAS	FHOD1	G12D	de-stabilizating
HRAS	FHOD1	G13D	de-stabilizating
HRAS	FHOD1	Q61H	stabilizing
HRAS	RALGDS	G12D	stabilizing
HRAS	RALGDS	G13D	stabilizing
HRAS	RALGDS	A59G	stabilizing
KRAS	RALGDS	G13D	stabilizing

Several mutants were predicted to stabilize complexes that promote tumorigenesis. A59G and G12D mutations on HRAS and G13D mutations on both HRAS and KRAS are predicted to stabilize interactions with RALGDS. RALGDS is an activator of HRAS and KRAS, thus stabilization of this interaction should increase the amount of RAS protein in the active state, consistent with RAS pathway activation during tumorigenesis. Additionally, we observed that A146T is predicted to stabilize the HRAS – BCL2 interaction that inhibits RAS-induced apoptosis [63].

**Fig. 6:**
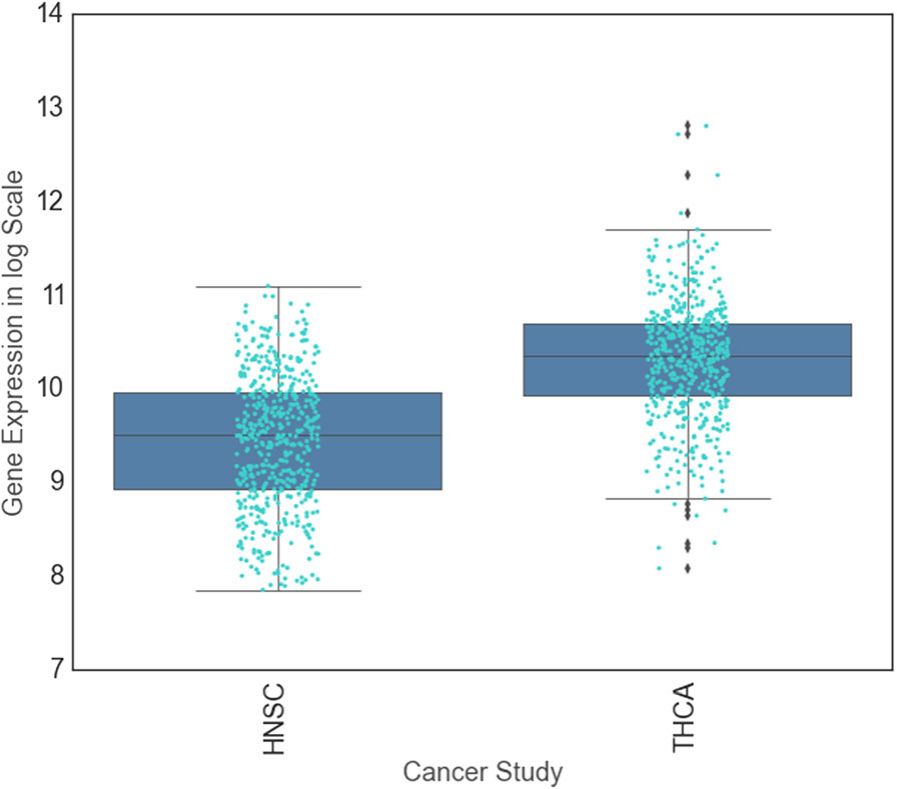
Tumor type specific expression of FHOD1. Increased expression of FHOD1 (Student’s t-test *p*-value <2 × 10^−16^) in thyroid samples compared to head & neck samples. This graph was produced using the gene expression data available via cBioPortal [92]

Interestingly, interactions with FHOD1 were destabilized by some mutants, and stabilized by others. FoldX predicted that G12 and G13 mutations de-stabilize HRAS-FHOD1 binding, while Q61 mutations stabilize it. These conflicting predictions suggest that FHOD1 may promote oncogenic RAS signaling in some settings and interfere with it in others. To further investigate this possibility, we investigated FHOD1 expression in two settings: head and neck cancer and thyroid cancer and thyroid cancer. In head and neck cancer, HRAS mutations are biased toward G12 and G13, whereas in thyroid cancer, HRAS mutations are most commonly observed at Q61. We observed that expression of FHOD1 is generally higher in thyroid tumors than in head and neck cancers, suggesting that Q61 may be more advantageous in thyroid cancer in part because of tumor type specific FHOD1 activity (Fig. 6, Additional file 4: Figure S4). Alternatively, this pattern might suggest that a shift in HRAS binding preference from FHOD1 to another partner such as RALGDS is favorable for head and neck cancer but less favorable for thyroid cancer.

### Inferring RAS co-complexes from predicted binding residues

Using the map of residues that mediate interactions with different RAS binding proteins, it is possible to predict higher order protein complexes that could form. We first eliminated any possible complexes including RAS interaction partner pairs that competed for interface residues on RAS, then used a steric clash filter to determine whether the remaining partners could bind RAS at the same time without interfering with each other (see the Methods; Table 3). This resulted in a list of 38 putative multi-protein complexes composed of RAS first-degree interactors and RAS proteins (Table 3). We found support for several of these predicted complexes in the literature. These RAS multi-protein complexes may help explain how RAS binding partners interact to regulate RAS signaling.

**Table 3:** The list of 38 multi-protein complexes that consist of RAS first-degree interactors and RAS proteins

RAS Multi-Protein Complex Candidates	
KRAS + (ARAF || RAF1 || BRAF || RALGDS) + CALM	
HRAS + (ERBB2IP || SRC || RABGEF1 || RARRES3 || LGALS1 || BCL2) + FHOD1	
HRAS + (SRC || RARRES3 || LGALS1) + PLCE	
HRAS + (RARRES3 || LGALS1) + ERBB2IP	
HRAS + (RARRES3) + SOS1	
HRAS + (GRB14 || RALGDS || ARAF || NF1 || RASA1) + SRC	
HRAS + (RALGDS || NF1) + RABGEF1	
HRAS + (GRB14 || RALGDS || ARAF || NF1 || PIK3CG || RAF1 || RASA1 || BCL2) + RARRES3	
HRAS + (GRB14 || RALGDS || ARAF || NF1 || PIK3CG || RAF1 || RASA1) + LGALS1	

Nussinov et al. [2] recently proposed the existence of a multi-protein complex composed of KRAS, PI3K and CALM. According to their model, CALM both sequesters KRAS from the PM and activates PI3Kα. The experimentally validated RAS interactions we used to build our models did not include an interaction between KRAS and PI3Kα, however, one predicted multi-protein complex model included KRAS, CALM and RAF1. Both RAF1 and PI3K bind to RAS using an RBrD. In addition, KRAS has been shown to bind to both RAF and CALM proteins at the same time and the binding of KRAS to CALM is independent from RAF [8].

Galectin-1 (LGALS1) can directly bind HRAS [37] and diverts RAS signals to RAF1 at the expense of PI3K [64]. According to our co-complex models, LGALS1 and switch region-binding partners such as RAF and PIK3CG can bind HRAS at the same time without causing steric clashes. However, it is known that RAS mediated activation of PIK3CG is dependent on recruitment of the PIK3CG catalytic subunit to the PM by the regulatory subunit PIK3R5 [65, 66]. Our models suggest that LGALS1 occludes the binding site for the larger PIK3CG-PIK3R5 complex, thereby favoring LGALS1-HRAS-RAF1 complex formation.

### Farnesyltransferase and HRAS in complex

**Fig. 7:**
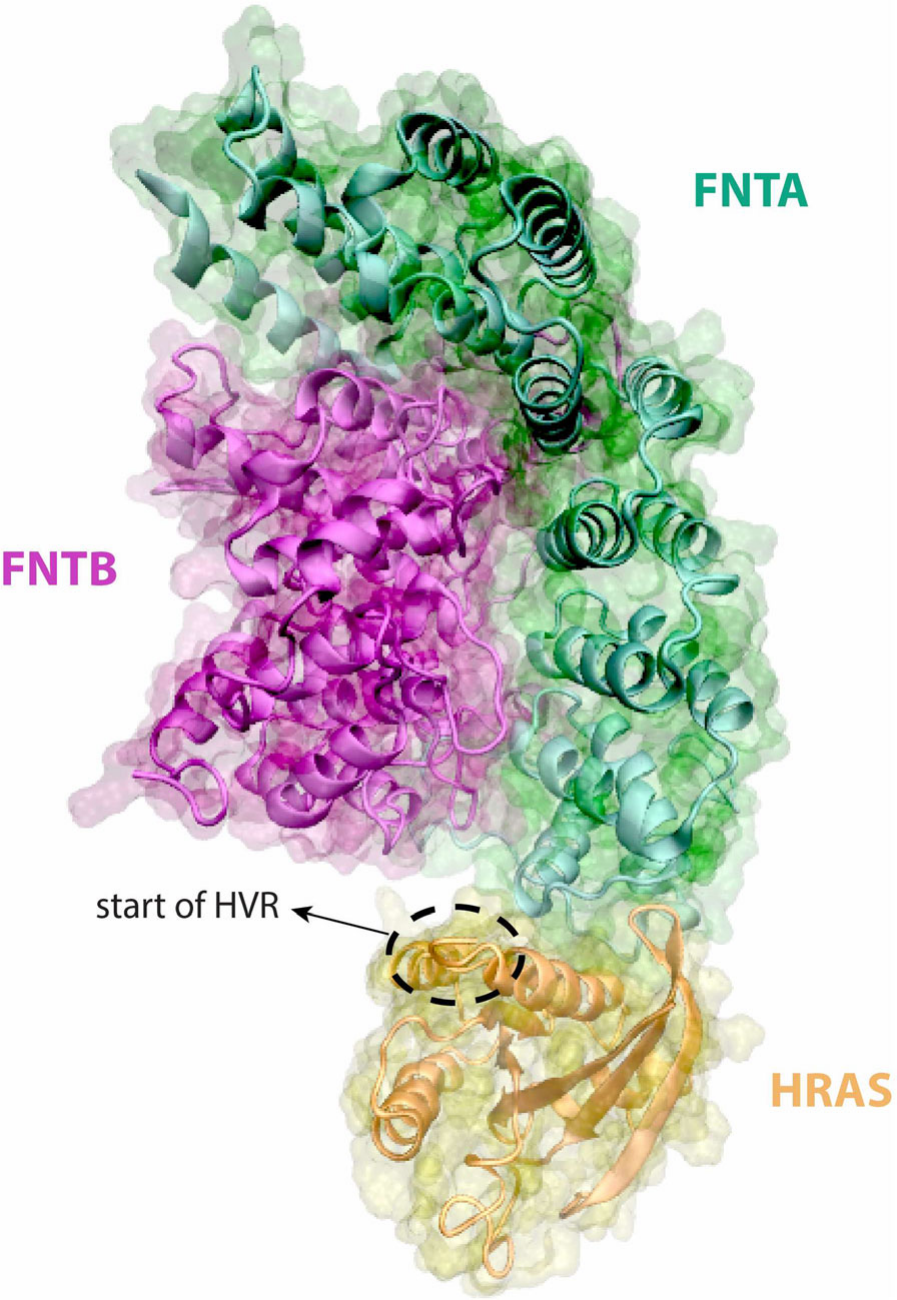
Putative Farnesyltransferase and HRAS complex

Finally, we investigated the RAS CAAX motif modifier Farnesyltransferase (FNT), which is responsible for the attachment of RAS proteins to the PM [67]. The FNT complex, which consists of FNTA and FNTB proteins, binds directly to RAS and modifies the CAAX Cys residue with a 15-carbon farnesyl lipid [67]. Although the structure of the FNT complex is available in the PDB (PDBID: 2H6F), the 3D structure of its interaction with RAS is not available. We therefore modeled the interaction between FNT and HRAS using PRISM [26] (binding energy = −11.0) and observed that FNT most likely binds via the allosteric/HVR regions on the RAS surface, using amino acids 4, 44–48, 157, 158, 161, 164, 165 and 168 (Fig. 7). Our model further predicts that the HRAS-FNT complex is capable of simultaneously binding SOS1, but not ERBB2IP, LGALS1, RABGEF1, RARRES3, or SRC. LGALS1 interaction with HRAS is known to be mutually exclusive to FNT interaction. These proteins are sequential regulators of HRAS localization. LGALS1 recognizes the farnesyl group on HRAS [68] after it has been modified by FNT [67].

## Discussion

RAS mutations are found in almost 30% of tumors, making these proteins an attractive target for therapy. However, targeting RAS therapeutically has proven challenging, due in part to incomplete knowledge of RAS protein interactions. Most therapies have focused on targeting effector interactions with the switch regions [18, 19, 69, 70], however new evidence suggests that therapies targeted to the allosteric region [71] may succeed where other attempts have failed. Spencer-Smith et al. developed a synthetic protein (NS1) that binds to HRAS and KRAS between residues 122 and 166, located in the allosteric region and inhibits oncogenic RAS signaling.

Understanding of RAS protein interaction interfaces is still largely restricted to common effectors and proteins with RBrDs, which comprise only ~40% of reported RAS binding proteins. Using structural modeling techniques, we were able to investigate protein complex formation between RAS proteins and the experimentally implicated RAS isoform-specific interaction network (Fig. 4). Of these, 45 proteins did not have a RAS binding domain, including post-translational modifiers such as FNTA and FNTB, scaffolding proteins such as PDE6D and LGALS1, ubiquitination pathway proteins such as UBC and BRAP and proteins that are reported to impair oncogenic RAS such as RARRES3 and RABGEF1. Our binding site predictions suggest that multiple RAS binding partners without RBrDs interact with RAS proteins via the allosteric and HVR regions. In particular we found 7 interaction partners (1 KRAS and 6 HRAS) that are predicted to bind the allosteric regions in the same region as NS1. Interestingly, for 37 reported RAS interaction partners with available structural data, PRISM could not find a viable binding site. These could represent false interactions, or could be false negative predictions resulting from crystal structures that are incomplete or fail to capture a binding-essential conformational state.

The importance of cellular localization as a determinant of RAS signaling is becoming increasingly apparent. It has now been demonstrated that RAS signaling takes place in distinct cytosolic regions including the PM [72], the ER [73] and the Golgi complex [74, 75]. Switch-region based interactions with common oncogenic effectors are reported to occur predominantly when RAS is located at the PM [72], whereas many of the interactions that we mapped to allosteric and/or HVR regions have been reported in the cytoplasm [36, 40, 76]. RAS dimerization is mediated at least in part through the allosteric domain [28, 71]. One possible explanation for the apparent correlation between RAS localization and binding region could lie in a propensity for PM localized RAS to dimerize, thereby occluding the allosteric region. Notably, several supposedly cytosolic interactions, such as BCL2 [76], RARRES3 [40] and LGALS1 [36] have clear significance for oncogenic RAS signaling. Schmick et al. [77] speculated that changes in the concentration of RAS at the PM versus at organelle endomembranes could act as a switch for RAS signaling. Interestingly, we find that multiple proteins predicted to bind the RAS allosteric/HVR region are determinants of RAS localization.

In addition to localization, the proportion of RAS bound in particular complexes could have implications for oncogenic signaling and could help explain the activities of particular therapeutic inhibitors. Using our model of RAS isoform interaction interfaces, we were able to predict competitive and synergistic binding of RAS interaction partners. This allowed us to narrow a large combinatorial space of interactions to 38 likely RAS multi-protein complexes that included HRAS-RAF-LGALS1, HRAS-RAF-RARRES3 and KRAS-RAF-CALM. Interestingly, both LGALS1 and Farnesyltransferase use overlapping binding regions on RAS, suggesting mutual exclusivity of these two entities. Given that they have distinct roles in RAS membrane association, this finding may guide studies of the dynamics of RAS membrane association.

Of 6 proteins in the RAS interaction network that are targeted by FDA approved drugs (RAF1, SRC, BRAF, AGTR1, PSMB2, BCL2) [22], two were implicated as binding RAS residues outside of the switch regions, BCL2 and SRC. PRISM did not predict interfaces for AGTR1 and PSMB2, suggesting that these proteins do indeed bind RAS, but they are unlikely to do so via the switch region. Interestingly, the small molecule inhibitor, ABT-263, that targets BCL2, is reported to cause synthetic lethality in KRAS mutant tumors when combined with Trametinib, a MEK inhibitor [78]. Although we did not predict an interaction for BCL2 with KRAS, BCL2 was predicted to bind to both NRAS and HRAS. Our predictions further suggest that BCL2 and RAS effectors involved in MEK signaling, BRAF, ARAF, RAF1 are unlikely to bind to RAS isoforms simultaneously. This knowledge may be helpful for designing follow up experimental studies to further investigate the mechanisms resulting in the synthetic lethality of the ABT-263 Trametinib combination in mutant RAS cells.

Observed patterns of somatic mutations in RAS isoforms across tumor types suggest that biological context determines the advantage of particular mutations. A compelling possible explanation for the specificity of somatic RAS mutations is that tissue level patterns of gene expression results in different ratios of RAS interaction partners, and these differences modify the functionality to RAS mutations. Studying the implications of 5 common RAS mutations for interactions, we found evidence that different RAS binding partners could be affected in different ways by the same mutations. For example, G12 and G13 mutations favored RGALS1 binding, but impaired FHOD1 binding. Interestingly, the HRAS-FHOD1 interaction is subject to both stabilizing (Q61) and destabilizing (G12 and G13) mutations, however these events were associated with distinct tumor types. This suggests that the prevalence of particular RAS mutations in a tumor type may reflect the protein interactions that best contribute to oncogenic RAS signaling in that tumor.

## Conclusions

We used structural modeling to map RAS binding interfaces and infer possible multi-protein complexes. Analysis of the resulting binding site map suggests that the allosteric region of RAS may play a more important role in RAS signaling than previously thought, and this role may go beyond RAS dimerization. Protein interactions utilizing the RAS allosteric region appear to be important for RAS localization, which is an important determinant of oncogenic RAS signaling. Further studies are needed to investigate the implications of this new aspect of RAS signaling in cancer.

## Methods

### RAS protein interaction network

We gathered experimentally validated HRAS, KRAS and NRAS protein interactions available in the STRING and PDB databases as of October 2015. We considered PPIs from STRING with confidence score greater than or equal to 0.4 (medium confidence) and manually checked each article associated with the given interaction. We discarded PPIs that had vague implications of physical interaction (such as the experiments that only used HRAS but claimed that the interactions were present for all 3 isoforms) from our dataset. Our network consists of 103 RAS protein interactions with 77 unique proteins; including 68 HRAS, 19 KRAS and 16 NRAS interactors (Fig. 2, Table 4). There is also evidence describing KRAS, NRAS and HRAS dimerization in the literature [28, 29]. Edges in the network represent the presence of strong published evidence supporting a physical interaction. We note that there is a strong bias toward probing RAS interactions using HRAS likely due to experimental convenience, and this is reflected in our network.

**Table 4:** Experimentally validated isoform specific RAS interactions

HRAS	AGTR1	HRAS	RABGEF1	KRAS	ARAF
HRAS	ARAF	HRAS	RAF1	KRAS	BCL2
HRAS	BCL2	HRAS	RALGDS	KRAS	BRAF
HRAS	BRAF	HRAS	RAP1GDS1	KRAS	BTRC
HRAS	BRAP	HRAS	RAPGEF1	KRAS	CALM1
HRAS	BTRC	HRAS	RAPGEF2	KRAS	EPB42
HRAS	CAV1	HRAS	RARRES3	KRAS	FNTA
HRAS	DAB2IP	HRAS	RASA1	KRAS	FNTB
HRAS	DGKZ	HRAS	RASGRF1	KRAS	PDE6D
HRAS	ERBB2IP	HRAS	RASGRP1	KRAS	PGGT1B
HRAS	FHOD1	HRAS	RASIP1	KRAS	PIK3CG
HRAS	FNTA	HRAS	RASSF1	KRAS	RAF1
HRAS	FNTB	HRAS	RASSF2	KRAS	RALGDS
HRAS	GPSM2	HRAS	RASSF5	KRAS	RAP1GDS1
HRAS	GRB14	HRAS	RGL1	KRAS	RASGRP2
HRAS	GRB2	HRAS	RGL2	KRAS	RASSF2
HRAS	IRAK1	HRAS	RGL4	KRAS	RASSF5
HRAS	ITSN1	HRAS	RGS12	KRAS	SHOC2
HRAS	LATS2	HRAS	RGS14	KRAS	UBC
HRAS	LGALS1	HRAS	RIN1	NRAS	BCL2
HRAS	MAPK8	HRAS	RIT2	NRAS	BRAF
HRAS	MLLT4	HRAS	RNF115	NRAS	CD2AP
HRAS	NEDD8	HRAS	SH3KBP1	NRAS	GRB10
HRAS	NF1	HRAS	SHOC2	NRAS	GRB14
HRAS	PDE6D	HRAS	SLC9A3R2	NRAS	PIK3CG
HRAS	PDGFB	HRAS	SNX17	NRAS	RAF1
HRAS	PIK3CA	HRAS	SOS1	NRAS	RALGDS
HRAS	PIK3CD	HRAS	SPRY2	NRAS	RAP1GDS1
HRAS	PIK3CG	HRAS	SRC	NRAS	RASGRP2
HRAS	PLCE1	HRAS	TIAM1	NRAS	RGL1
HRAS	PPP1R13B	HRAS	TP53	NRAS	RNASEH2B
HRAS	PRKCI	HRAS	TP73	NRAS	SHOC2
HRAS	PSMB2	HRAS	TPR	NRAS	SPATS2
HRAS	RABAC1	HRAS	TTC1	NRAS	UBC
		HRAS	UBC	NRAS	VAT1

### RAS binding related domains (RBrDs)

Protein domain data was downloaded from the PFAM database in February 2015. We annotated any protein hosting a RAS binding domain (such as RBD, RA, PI3K_RBD, etc.), GAP domain (such as RAS_GAP) or GEF domain (such as RasGEF, RasGEF_N, RhoGEF, etc.) as having a RAS binding related domain (RBrD).

### Structural data available for RAS interactions

Among the 81 proteins in our network (including the RAS proteins), 50 had some structural data in PDB. There were 7 RAS complexes in the PDB (HRAS - RAF1 (PDB ID: 3KUD), HRAS – PLCE (PDB ID: 2C5L), HRAS – RASA1 (PDB ID: 1WQ1), HRAS – PIK3CG (PDB ID: 1HE8), HRAS – SOS1 (PDB ID: 1NVV), HRAS – GRB14 (PDB ID: 4 K81) and KRAS-ARAF (PDB ID: 2MSE)). One PDB structure displayed a biological homodimer of HRAS (PDB ID: 3lo5, chains: A and C) but no equivalent structure was found for KRAS or NRAS. We used PISA [79] to differentiate biological homodimers from crystallographic artifacts.

### Building structural models of RAS interactions

We aligned PDB structures to discard redundant PDB chains but retained distinct conformations/domains of a given protein. We used TM-align [80] for the structural comparison and considered two PDB chains to be redundant if they received TM-scores larger than 0.5 and had an RMSD under 2.5 Å. We clustered redundant PDB chains together and selected the chain with largest resolution and longest sequence as the representative of the group. On average we retained 2.8 PDB chains per protein (Table 5).

**Table 5:** Representative PDB chains for each protein. The Entrez ID of the corresponding gene is provided

Entrez Gene ID	Representative PDB Chain(s) [PDB ID_Chain]	Entrez Gene ID	Representative PDB Chain(s) [PDB ID_Chain]
369	1WXM_A	5921	1WER_A 2GSB_A 2GQI_A
596	1G5M_A	6002	2EBZ_A 2KV8_A
673	3SKC_B 2L05_A 3NY5_A	6453	1KI1_B 2KHN_A 4IIM_A
808	1NKF_A 2JZI_A 2K0E_A 2KNE_A 2L53_A 2LGF_A 2M0K_A 2M55_A 2MG5_A 3O77_A 4DCK_B 1XFZ_O 3O78_A 4DJC_A 1ZUZ_A 2W73_B	6654	1AWE_A 1DBH_A 1XD4_A 1XDV_A 3KSY_A 1XD4_B 1Q9C_D
2339	2H6F_A	6714	4F5B_A 3VRO_B 3ZMP_C 3ZMP_D 3ZMQ_C 1Y57_A 4HXJ_A 1YOJ_A
2342	2H6F_B	7074	2D8I_A 4K2P_A
2885	1GFC_A 1FHS_A 1GRI_A	7157	1A1U_A 1H26_E 1JSP_A 1MA3_B 2B3G_B 2GS0_B 2K8F_B 2L14_B2LY4_B 2YDR_P 3TG5_B 1OLG_A 1DT7_X 3Q05_A 4BUZ_P 1YC5_B 3DAC_B 2F1X_B 2H4H_B 2FOJ_B
2887	1NRV_A 3HK0_A	7161	1COK_A 2KBY_A 3VD0_K
2888	2AUG_B 2AUH_B 4K81_A	7316	1CMX_D 3OFI_C 3OFI_D 3B0A_D
3265	6Q21_A	8945	1P22_A 2P64_B
3845	1D8D_P 1KZP_C 1KZO_C 4DSO_A 2MSE_B 2MSD_B 2MSC_B	9351	2D11_E 2HE4_A
3956	3W58_C	9784	3LUI_C 4GXB_A
4301	1XZ9_A 2EXG_A	10,125	4L9M_A 4L9U_A
4738	3DBH_I	10,235	2MA2_A
4763	1NF1_A 3P7Z_A	10,253	3BUM_A
4893	3CON_A	10,636	2JNU_A 2XNS_C
5147	3T5I_A	11,186	2KZU_B
5155	3MJG_B	23,607	2FEI_A 3LK4_0
5290	2ENQ_A 3HHM_A	27,342	2OT3_A 2C7N_I
5294	3IBE_A	29,109	3DAD_A
5584	1VD2_A 3A8X_A	29,899	3SF4_A
5599	3O2M_A	30,011	2O2O_A
5894	4G0N_B 4IHL_P 1FAQ_A 4FJ3_P 3OMV_A 1C1Y_B	51,196	2BYE_A 2BYF_A
5900	3KH0_B	55,914	2H3L_A 2QBW_A 3CH8_A
5920	2LKT_A	79,621	3PUF_H 3P87_H
		83,593	4LGD_G

We used PRISM [26] to identify physical binding configurations (complexes) between proteins. Previously, PRISM has been successfully applied for modeling PPIs from various pathways including apoptosis [81], ubiquitination [82], MAPK [83], the Toll-like receptor pathways [84], and for identifying possible drug interactions causing off-target effects [85]. In order to assess the performance of rigid-body prediction algorithms, docking benchmarks are widely used. PRISM was capable of building near native models for 87 out of 88 cases in the docking benchmark [27].

Protein complexes predicted by PRISM becomes more energetically favorable as the energy score decreases. The PRISM server reports only the models with energy score smaller than 0, and a threshold of −10 has been used previously in the PRISM literature [85, 86]. PRISM v2.0 complexes receiving an energy score of −10 or smaller correspond to the 65th percentile of all models generated by the webserver as of 01/05/2017. For this work, we considered complexes receiving a score of −20 or smaller, corresponding to the 40th percentile (Table 1). At this threshold, we obtained 20 models covering 17 PPIs, with energy scores ranging from −52.35 to −20 (Fig. 3). PRISM has previously reported predictions as extreme as −829. If multiple configurations were implicated for the same RAS partner, the predicted complex with the lowest binding energy was used.

We ran PRISM with multiple distinct KRAS and HRAS chains, but only two chains generated complex predictions: 6q21 (A chain for HRAS) and 4dso (A chain for KRAS). There was only one PDB chain available for NRAS (3con:A) and it was missing the residues between 60 and 72. We therefore generated a complete model for NRAS via Swiss-Model [87] server.

For selected interfaces, PRISM predictions were compared to those of three other tools designed to predict protein complexes pyDock [54], ZDOCK [55] and COTH [56]. Predictions by these tools were obtained from their respective web servers by entering PDB IDs, and the top scoring predicted complex was selected.

### Interface region extraction

We used the consensus set of KFC2 [88] and HotPoint [89] server predictions to extract the specific amino acid positions of the interface region from PRISM predicted complexes. The structures of the domains containing interface regions for proteins predicted to bind outside of the Switch domain were compared using VAST [90] via the NCBI structure database (https://www.ncbi.nlm.nih.gov/structure).

### Steric clash filter

To determine whether RAS binding partners could bind RAS simultaneously, we used structural data to investigate whether simultaneous binding would create steric clashes. To do this we aligned 11 HRAS/4 KRAS complexes (both experimentally derived (Additional file 5: Figure S5) and predicted RAS complexes) to a reference HRAS/KRAS protein (HRAS PDB ID: 6Q21:A; KRAS PDB ID: 4DSO:A) via the “Profit” [91] protein aligner. Then, we calculated the Euclidean distance between the atoms of each protein in the complex. If the distance between any atom pair was less than 1 Å, we assumed the RAS interaction partners would result in steric clashes and therefore could not simultaneously bind RAS. We note that we used the HRAS-RAF1 complex available in PDB instead of the predicted complex generated by PRISM to evaluate steric clashes between RAF1 and other HRAS binding partners.

### Simulating the effects of mutations on protein-protein interactions

RAS hotspot mutations and tissue specificities were obtained from the literature [57, 62]. We used FoldX [60] to simulate the effects of mutations on PRISM-predicted protein complexes. For this purpose we calculated the binding affinity change after a mutation was introduced. As suggested in the FoldX manual, we assumed that free energy changes with a magnitude smaller than 0.5 were neutral. Larger positive free energy changes correspond to a destabilizing effect on complex formation, whereas larger negative free energy changes correspond to a stabilizing effect on complex formation.

## Additional files

Additional file 1: Fig. S1. Map of predicted HRAS-RARRES3 interface residues. We have used the most favorable (lowest binding energy score) predictions for HRAS (PDB ID: 6q21A) and RARRES3 (PDB ID: 2lktA) reported by ZDOCK, pyDock, COTH and PRISM. (PNG 147 kb)

Additional file 2: Fig. S2. Map of predicted KRAS-CALM interface residues. We have used the most favorable (lowest binding energy score) predictions for KRAS (PDB ID: 4dsoA) and CALM (PDB ID: 1zuzA) reported by ZDOCK, pyDock, COTH and PRISM. (PNG 147 kb)

Additional file 3: Fig. S3. Map of predicted HRAS-RAF1 interface residues. We have used the most favorable (lowest binding energy score) predictions for HRAS (PDB ID: 6q21A) and RAF1 (PDB ID: 4g0nB) reported by ZDOCK, pyDock, COTH and PRISM. (PNG 148 kb)

Additional file 4: Fig. S4. Pancancer FHOD1 gene expression distribution. This graph was produced using the gene expression data available via cBioPortal [92]. (PNG 56 kb)

Additional file 5: Fig. S5. Map of known RAS interfaces. Interaction interface residues from the experimentally derived HRAS complexes and RAS dimerization interfaces according to the literature. (PNG 199 kb)

## References

[1] Stephen AG, Esposito D, Bagni RK, McCormick F (2014). Dragging ras back in the ring. Cancer Cell.

[2] Nussinov R, Muratcioglu S, Tsai CJ, Jang H, Gursoy A, Keskin O (2016). K-Ras4B/calmodulin/PI3Kalpha: a promising new adenocarcinoma-specific drug target?. Expert Opin Ther Targets.

[3] Vigil D, Cherfils J, Rossman KL, Der CJ (2010). Ras superfamily GEFs and GAPs: validated and tractable targets for cancer therapy?. Nat Rev Cancer.

[4] Bar-Sagi D (2001). A Ras by any other name. Mol Cell Biol.

[5] Santos E (2014). Dimerization opens new avenues into Ras signaling research. Sci Signal.

[6] Abraham SJ, Nolet RP, Calvert RJ, Anderson LM, Gaponenko V (2009). The hypervariable region of K-Ras4B is responsible for its specific interactions with calmodulin. Biochemistry.

[7] Jang H, Muratcioglu S, Gursoy A, Keskin O, Nussinov R. Membrane-associated Ras dimers are isoform-specific: K-Ras dimers differ from H-Ras dimers. Biochem J. 2016; doi:10.1042/BCJ20160031. 10.1042/BCJ20160031 PMC7830773 27057007

[8] Villalonga P, Lopez-Alcala C, Bosch M, Chiloeches A, Rocamora N, Gil J (2001). Calmodulin binds to K-Ras, but not to H- or N-Ras, and modulates its downstream signaling. Mol Cell Biol.

[9] Cancer Genome Atlas N (2015). Comprehensive genomic characterization of head and neck squamous cell carcinomas. Nature.

[10] Cancer Genome Atlas N (2015). Genomic classification of Cutaneous melanoma. Cell.

[11] Witkiewicz AK, McMillan EA, Balaji U, Baek G, Lin WC, Mansour J (2015). Whole-exome sequencing of pancreatic cancer defines genetic diversity and therapeutic targets. Nat Commun.

[12] Kempf E, Rousseau B, Besse B, Paz-Ares L (2016). KRAS oncogene in lung cancer: focus on molecularly driven clinical trials. Eur Respir Rev.

[13] Bryant KL, Mancias JD, Kimmelman AC, Der CJ (2014). KRAS: feeding pancreatic cancer proliferation. Trends Biochem Sci.

[14] Chavan TS, Jang H, Khavrutskii L, Abraham SJ, Banerjee A, Freed BC (2015). High-affinity interaction of the K-Ras4B Hypervariable region with the Ras active site. Biophys J.

[15] Shieh A, Ward AF, Donlan KL, Harding-Theobald ER, Xu J, Mullighan CG (2013). Defective K-Ras oncoproteins overcome impaired effector activation to initiate leukemia in vivo. Blood.

[16] Cox AD, Fesik SW, Kimmelman AC, Luo J, Der CJ (2014). Drugging the undruggable RAS: mission possible?. Nat Rev Drug Discov.

[17] Maurer T, Garrenton LS, Oh A, Pitts K, Anderson DJ, Skelton NJ (2012). Small-molecule ligands bind to a distinct pocket in Ras and inhibit SOS-mediated nucleotide exchange activity. Proc Natl Acad Sci U S A.

[18] Ostrem JM, Peters U, Sos ML, Wells JA, Shokat KM (2013). K-Ras(G12C) inhibitors allosterically control GTP affinity and effector interactions. Nature.

[19] Shima F, Yoshikawa Y, Ye M, Araki M, Matsumoto S, Liao J (2013). In silico discovery of small-molecule Ras inhibitors that display antitumor activity by blocking the Ras-effector interaction. Proc Natl Acad Sci U S A.

[20] Sun Q, Burke JP, Phan J, Burns MC, Olejniczak ET, Waterson AG (2012). Discovery of small molecules that bind to K-Ras and inhibit Sos-mediated activation. Angew Chem Int Ed Engl.

[21] Athuluri-Divakar SK, Vasquez-Del Carpio R, Dutta K, Baker SJ, Cosenza SC, Basu I (2016). A small molecule RAS-mimetic disrupts RAS Association with Effector proteins to block signaling. Cell.

[22] Ponten F, Jirstrom K, Uhlen M (2008). The human protein Atlas--a tool for pathology. J Pathol.

[23] Nussinov R, Tsai CJ, Muratcioglu S, Jang H, Gursoy A, Keskin O (2015). Principles of K-Ras effector organization and the role of oncogenic K-Ras in cancer initiation through G1 cell cycle deregulation. Expert Rev Proteomics.

[24] Jaumot M, Yan J, Clyde-Smith J, Sluimer J, Hancock JF (2002). The linker domain of the ha-Ras hypervariable region regulates interactions with exchange factors, Raf-1 and phosphoinositide 3-kinase. J Biol Chem.

[25] Berman H, Henrick K, Nakamura H (2003). Announcing the worldwide protein data Bank. Nat Struct Biol.

[26] Tuncbag N, Gursoy A, Nussinov R, Keskin O (2011). Predicting protein-protein interactions on a proteome scale by matching evolutionary and structural similarities at interfaces using PRISM. Nat Protoc.

[27] Tuncbag N, Keskin O, Nussinov R, Gursoy A (2012). Fast and accurate modeling of protein-protein interactions by combining template-interface-based docking with flexible refinement. Proteins.

[28] Lin WC, Iversen L, Tu HL, Rhodes C, Christensen SM, Iwig JS (2014). H-Ras forms dimers on membrane surfaces via a protein-protein interface. Proc Natl Acad Sci U S A.

[29] Guldenhaupt J, Rudack T, Bachler P, Mann D, Triola G, Waldmann H (2012). N-Ras forms dimers at POPC membranes. Biophys J.

[30] Buerger C, DeVries B, Stambolic V (2006). Localization of Rheb to the endomembrane is critical for its signaling function. Biochem Biophys Res Commun.

[31] Finn RD, Coggill P, Eberhardt RY, Eddy SR, Mistry J, Mitchell AL (2016). The Pfam protein families database: towards a more sustainable future. Nucleic Acids Res.

[32] Xu L, Lubkov V, Taylor LJ, Bar-Sagi D (2010). Feedback regulation of Ras signaling by Rabex-5-mediated ubiquitination. Curr Biol.

[33] Lee S, Tsai YC, Mattera R, Smith WJ, Kostelansky MS, Weissman AM (2006). Structural basis for ubiquitin recognition and autoubiquitination by Rabex-5. Nat Struct Mol Biol.

[34] Mattera R, Tsai YC, Weissman AM, Bonifacino JS (2006). The Rab5 guanine nucleotide exchange factor Rabex-5 binds ubiquitin (Ub) and functions as a Ub ligase through an atypical Ub-interacting motif and a zinc finger domain. J Biol Chem.

[35] Penengo L, Mapelli M, Murachelli AG, Confalonieri S, Magri L, Musacchio A (2006). Crystal structure of the ubiquitin binding domains of rabex-5 reveals two modes of interaction with ubiquitin. Cell.

[36] Belanis L, Plowman SJ, Rotblat B, Hancock JF, Kloog Y (2008). Galectin-1 is a novel structural component and a major regulator of h-ras nanoclusters. Mol Biol Cell.

[37] Paz A, Haklai R, Elad-Sfadia G, Ballan E, Kloog Y (2001). Galectin-1 binds oncogenic H-Ras to mediate Ras membrane anchorage and cell transformation. Oncogene.

[38] Rotblat B, Ehrlich M, Haklai R, Kloog Y (2008). The Ras inhibitor farnesylthiosalicylic acid (Salirasib) disrupts the spatiotemporal localization of active Ras: a potential treatment for cancer. Methods Enzymol.

[39] Tsai FM, Shyu RY, Jiang SY (2007). RIG1 suppresses Ras activation and induces cellular apoptosis at the Golgi apparatus. Cell Signal.

[40] Tsai FM, Shyu RY, Jiang SY (2006). RIG1 inhibits the Ras/mitogen-activated protein kinase pathway by suppressing the activation of Ras. Cell Signal.

[41] Tokumitsu Y, Nakano S, Ueno H, Niho Y (2000). Suppression of malignant growth potentials of v-Src-transformed human gallbladder epithelial cells by adenovirus-mediated dominant negative H-Ras. J Cell Physiol.

[42] van der Geer P, Wiley S, Gish GD, Pawson T (1996). The Shc adaptor protein is highly phosphorylated at conserved, twin tyrosine residues (Y239/240) that mediate protein-protein interactions. Curr Biol.

[43] Thornton C, Yaka R, Dinh S, Ron D (2003). H-Ras modulates N-methyl-D-aspartate receptor function via inhibition of Src tyrosine kinase activity. J Biol Chem.

[44] Fivaz M, Meyer T (2005). Reversible intracellular translocation of KRas but not HRas in hippocampal neurons regulated by Ca2+/calmodulin. J Cell Biol.

[45] Villalonga P, Lopez-Alcala C, Chiloeches A, Gil J, Marais R, Bachs O (2002). Calmodulin prevents activation of Ras by PKC in 3T3 fibroblasts. J Biol Chem.

[46] Sidhu RS, Clough RR, Bhullar RP (2003). Ca2+/calmodulin binds and dissociates K-RasB from membrane. Biochem Biophys Res Commun.

[47] Nussinov R, Tsai CJ, Chakrabarti M, Jang H (2016). A new view of Ras Isoforms in cancers. Cancer Res.

[48] Moreto J, Vidal-Quadras M, Pol A, Santos E, Grewal T, Enrich C (2009). Differential involvement of H- and K-Ras in Raf-1 activation determines the role of calmodulin in MAPK signaling. Cell Signal.

[49] DiSepio D, Ghosn C, Eckert RL, Deucher A, Robinson N, Duvic M (1998). Identification and characterization of a retinoid-induced class II tumor suppressor/growth regulatory gene. Proc Natl Acad Sci U S A.

[50] Thomas C, Strutt D (2014). Rabaptin-5 and Rabex-5 are neoplastic tumour suppressor genes that interact to modulate Rab5 dynamics in *Drosophila melanogaster*. Dev Biol.

[51] Berchtold MW, Villalobo A (2014). The many faces of calmodulin in cell proliferation, programmed cell death, autophagy, and cancer. Biochim Biophys Acta.

[52] Hsu YL, Wu CY, Hung JY, Lin YS, Huang MS, Kuo PL (2013). Galectin-1 promotes lung cancer tumor metastasis by potentiating integrin alpha6beta4 and Notch1/Jagged2 signaling pathway. Carcinogenesis.

[53] Irby RB, Yeatman TJ (2000). Role of Src expression and activation in human cancer. Oncogene.

[54] Cheng TM, Blundell TL, Fernandez-Recio J (2007). pyDock: electrostatics and desolvation for effective scoring of rigid-body protein-protein docking. Proteins.

[55] Pierce BG, Hourai Y, Weng Z (2011). Accelerating protein docking in ZDOCK using an advanced 3D convolution library. PLoS One.

[56] Mukherjee S, Zhang Y (2011). Protein-protein complex structure predictions by multimeric threading and template recombination. Structure.

[57] Prior IA, Lewis PD, Mattos C (2012). A comprehensive survey of Ras mutations in cancer. Cancer Res.

[58] Seo KY, Jelinsky SA, Loechler EL (2000). Factors that influence the mutagenic patterns of DNA adducts from chemical carcinogens. Mutat Res.

[59] Tormanen VT, Pfeifer GP (1992). Mapping of UV photoproducts within ras proto-oncogenes in UV-irradiated cells: correlation with mutations in human skin cancer. Oncogene.

[60] Schymkowitz J, Borg J, Stricher F, Nys R, Rousseau F, Serrano L (2005). The FoldX web server: an online force field. Nucleic Acids Res.

[61] Kiel C, Serrano L (2014). Structure-energy-based predictions and network modelling of RASopathy and cancer missense mutations. Mol Syst Biol.

[62] Chang MT, Asthana S, Gao SP, Lee BH, Chapman JS, Kandoth C (2016). Identifying recurrent mutations in cancer reveals widespread lineage diversity and mutational specificity. Nat Biotechnol.

[63] Chen CY, Faller DV (1996). Phosphorylation of Bcl-2 protein and association with p21Ras in Ras-induced apoptosis. J Biol Chem.

[64] Elad-Sfadia G, Haklai R, Ballan E, Gabius HJ, Kloog Y (2002). Galectin-1 augments Ras activation and diverts Ras signals to Raf-1 at the expense of phosphoinositide 3-kinase. J Biol Chem.

[65] Krugmann S, Hawkins PT, Pryer N, Braselmann S (1999). Characterizing the interactions between the two subunits of the p101/p110gamma phosphoinositide 3-kinase and their role in the activation of this enzyme by G beta gamma subunits. J Biol Chem.

[66] Stephens LR, Eguinoa A, Erdjument-Bromage H, Lui M, Cooke F, Coadwell J (1997). The G beta gamma sensitivity of a PI3K is dependent upon a tightly associated adaptor, p101. Cell.

[67] Rowinsky EK, Windle JJ, Von Hoff DD (1999). Ras protein farnesyltransferase: a strategic target for anticancer therapeutic development. J Clin Oncol.

[68] Yu X, Scott SA, Pritchard R, Houston TA, Ralph SJ, Blanchard H (2015). Redox state influence on human galectin-1 function. Biochimie.

[69] Lim SM, Westover KD, Ficarro SB, Harrison RA, Choi HG, Pacold ME (2014). Therapeutic targeting of oncogenic K-Ras by a covalent catalytic site inhibitor. Angew Chem Int Ed Engl.

[70] Wu X, Upadhyaya P, Villalona-Calero MA, Briesewitz R, Pei D (2013). Inhibition of Ras-Effector interaction by cyclic peptides. Medchemcomm.

[71] Spencer-Smith R, Koide A, Zhou Y, Eguchi RR, Sha F, Gajwani P, et al. Inhibition of RAS function through targeting an allosteric regulatory site. Nat Chem Biol. 2016; doi:10.1038/nchembio.2231. 10.1038/nchembio.2231 PMC5193369 27820802

[72] Prior IA, Hancock JF (2012). Ras trafficking, localization and compartmentalized signalling. Semin Cell Dev Biol.

[73] Arozarena I, Matallanas D, Berciano MT, Sanz-Moreno V, Calvo F, Munoz MT (2004). Activation of H-Ras in the endoplasmic reticulum by the RasGRF family guanine nucleotide exchange factors. Mol Cell Biol.

[74] Ahearn IM, Haigis K, Bar-Sagi D, Philips MR (2012). Regulating the regulator: post-translational modification of RAS. Nat Rev Mol Cell Biol.

[75] Bivona TG, Perez De Castro I, Ahearn IM, Grana TM, Chiu VK, Lockyer PJ (2003). Phospholipase Cgamma activates Ras on the Golgi apparatus by means of RasGRP1. Nature.

[76] Bivona TG, Quatela SE, Bodemann BO, Ahearn IM, Soskis MJ, Mor A (2006). PKC regulates a farnesyl-electrostatic switch on K-Ras that promotes its association with Bcl-XL on mitochondria and induces apoptosis. Mol Cell.

[77] Schmick M, Kraemer A, Bastiaens PI (2015). Ras moves to stay in place. Trends Cell Biol.

[78] Corcoran RB, Cheng KA, Hata AN, Faber AC, Ebi H, Coffee EM (2013). Synthetic lethal interaction of combined BCL-XL and MEK inhibition promotes tumor regressions in KRAS mutant cancer models. Cancer Cell.

[79] Krissinel E, Henrick K (2007). Inference of macromolecular assemblies from crystalline state. J Mol Biol.

[80] Zhang Y, Skolnick J (2005). TM-align: a protein structure alignment algorithm based on the TM-score. Nucleic Acids Res.

[81] Acuner Ozbabacan SE, Keskin O, Nussinov R, Gursoy A (2012). Enriching the human apoptosis pathway by predicting the structures of protein-protein complexes. J Struct Biol.

[82] Kar G, Keskin O, Nussinov R, Gursoy A (2012). Human proteome-scale structural modeling of E2-E3 interactions exploiting interface motifs. J Proteome Res.

[83] Kuzu G, Gursoy A, Nussinov R, Keskin O (2013). Exploiting conformational ensembles in modeling protein-protein interactions on the proteome scale. J Proteome Res.

[84] Guven Maiorov E, Keskin O, Gursoy A, Nussinov R (2013). The structural network of inflammation and cancer: merits and challenges. Semin Cancer Biol.

[85] Engin HB, Keskin O, Nussinov R, Gursoy A (2012). A strategy based on protein-protein interface motifs may help in identifying drug off-targets. J Chem Inf Model.

[86] Engin HB, Guney E, Keskin O, Oliva B, Gursoy A (2013). Integrating structure to protein-protein interaction networks that drive metastasis to brain and lung in breast cancer. PLoS One.

[87] Kiefer F, Arnold K, Kunzli M, Bordoli L, Schwede T (2009). The SWISS-MODEL repository and associated resources. Nucleic Acids Res.

[88] Zhu X, Mitchell JC (2011). KFC2: a knowledge-based hot spot prediction method based on interface solvation, atomic density, and plasticity features. Proteins.

[89] Tuncbag N, Gursoy A, Keskin O (2009). Identification of computational hot spots in protein interfaces: combining solvent accessibility and inter-residue potentials improves the accuracy. Bioinformatics.

[90] Gibrat JF, Madej T, Bryant SH (1996). Surprising similarities in structure comparison. Curr Opin Struct Biol.

[91] Martin ACRaP, C.T. http://www.bioinf.org.uk/software/profit/ Accessed 18 Feb 2015.

[92] Gao J, Aksoy BA, Dogrusoz U, Dresdner G, Gross B, Sumer SO (2013). Integrative analysis of complex cancer genomics and clinical profiles using the cBioPortal. Sci Signal.

