# Supplementary figures and images for: Modeling of RAS complexes supports roles in cancer for less studied partners
Source: BMC Biophys. 2017 Aug 11;10(Suppl 1):5. doi: 10.1186/s13628-017-0037-6 (PMC5558186; doi:10.1186/s13628-017-0037-6)

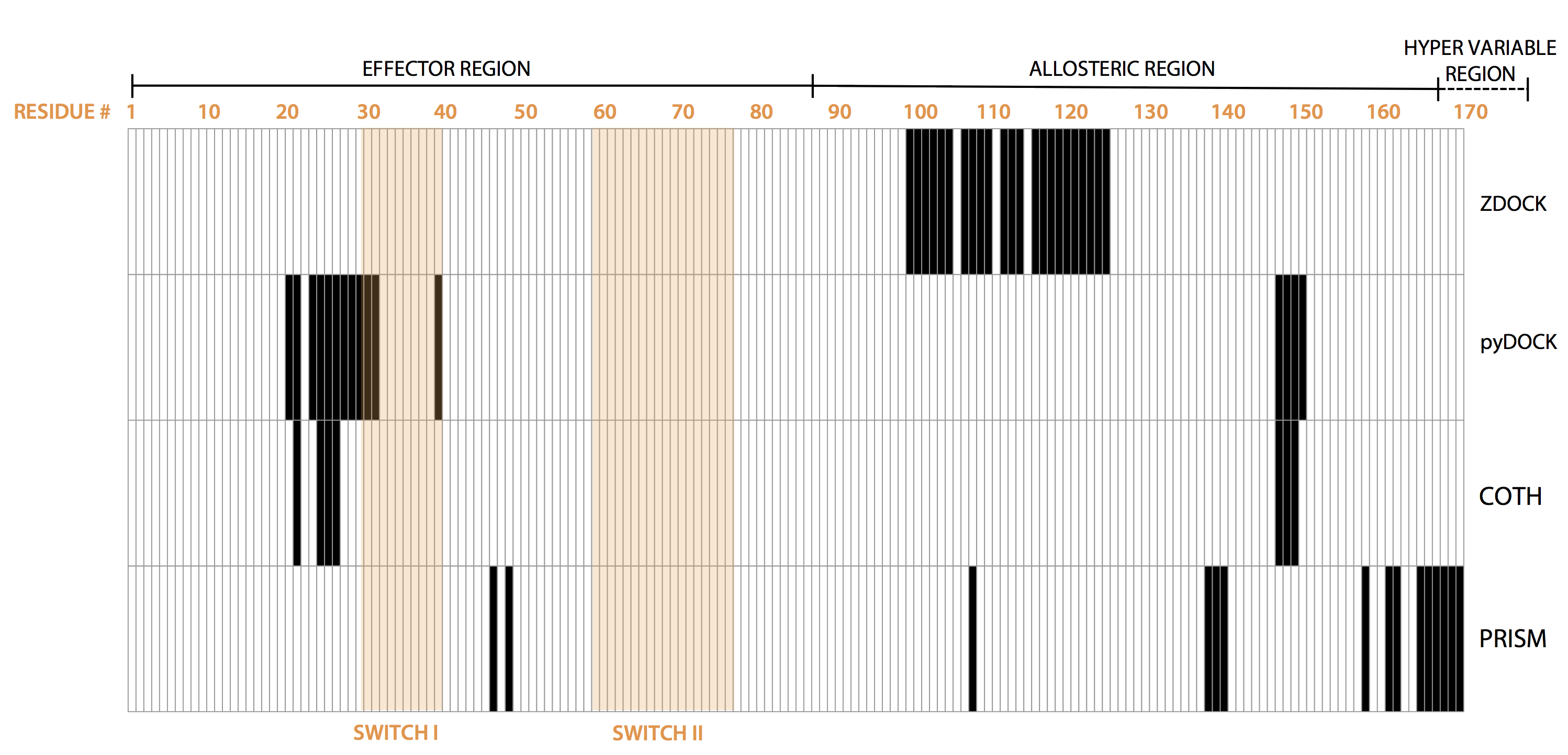

Supplement: Supplementary file 1 — Map of predicted HRAS-RARRES3 interface residues. We have used the most favorable (lowest binding energy score) predictions for HRAS (PDB ID: 6q21A) and RARRES3 (PDB ID: 2lktA) reported by ZDOCK, pyDock, COTH and PRISM. (PNG 147 kb) [file 13628_2017_37_MOESM1_ESM.png]

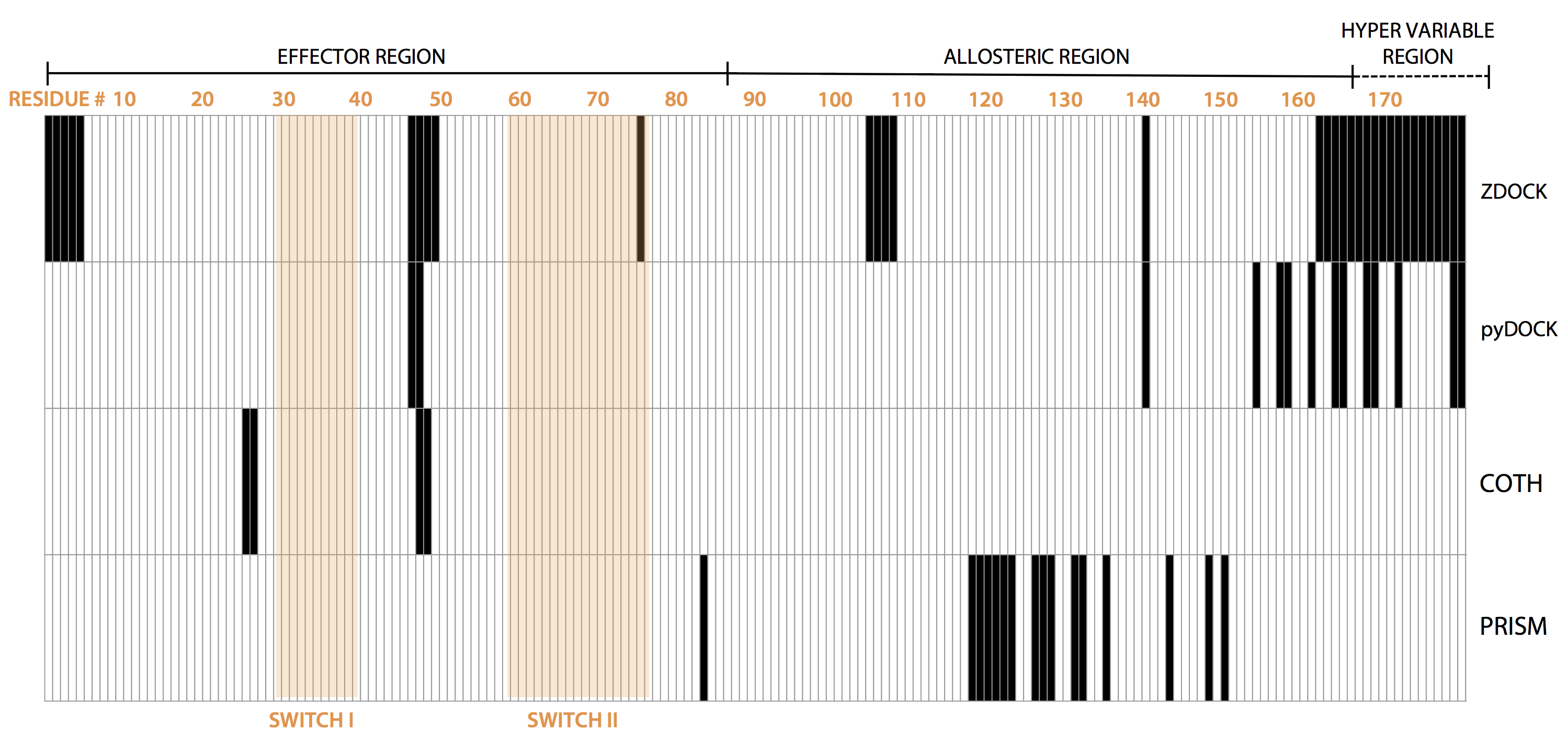

Supplement: Supplementary file 2 — Map of predicted KRAS-CALM interface residues. We have used the most favorable (lowest binding energy score) predictions for KRAS (PDB ID: 4dsoA) and CALM (PDB ID: 1zuzA) reported by ZDOCK, pyDock, COTH and PRISM. (PNG 147 kb) [file 13628_2017_37_MOESM2_ESM.png]

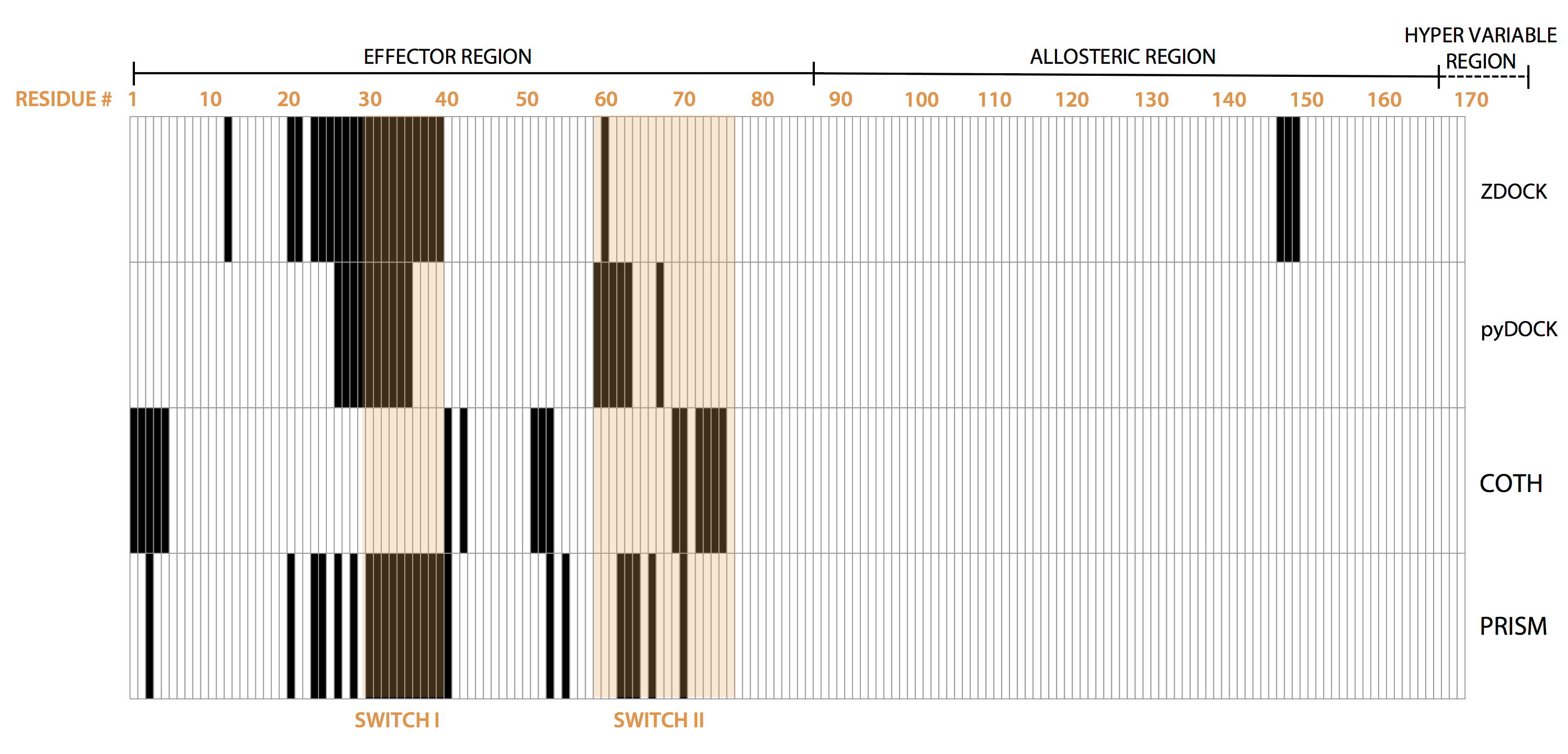

Supplement: Supplementary file 3 — Map of predicted HRAS-RAF1 interface residues. We have used the most favorable (lowest binding energy score) predictions for HRAS (PDB ID: 6q21A) and RAF1 (PDB ID: 4g0nB) reported by ZDOCK, pyDock, COTH and PRISM. (PNG 148 kb) [file 13628_2017_37_MOESM3_ESM.png]

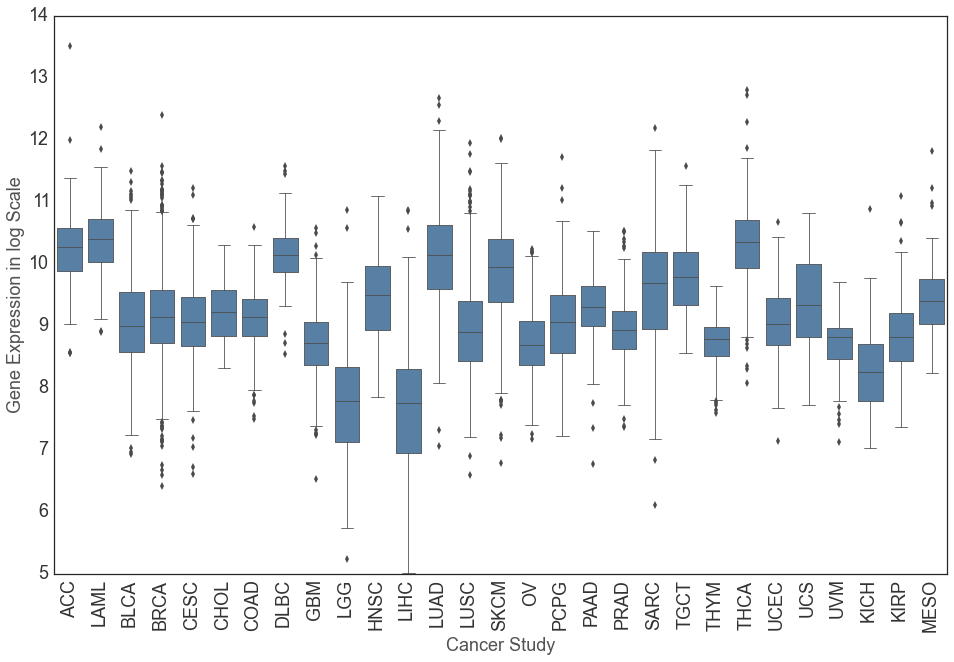

Supplement: Supplementary file 4 — Pancancer FHOD1 gene expression distribution. This graph was produced using the gene expression data available via cBioPortal [92]. (PNG 56 kb) [file 13628_2017_37_MOESM4_ESM.png]

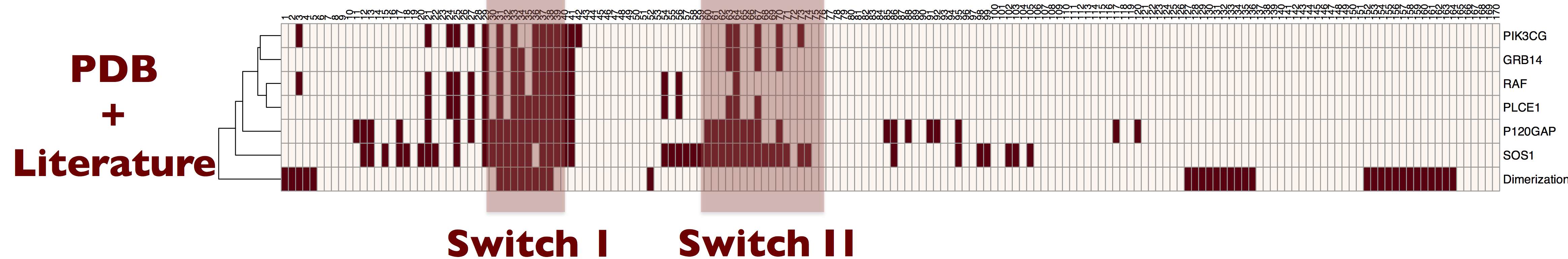

Supplement: Supplementary file 5 — Map of known RAS interfaces. Interaction interface residues from the experimentally derived HRAS complexes and RAS dimerization interfaces according to the literature. (PNG 199 kb) [file 13628_2017_37_MOESM5_ESM.png]
